# GnRH Neurons Provide Direct Input to Hypothalamic Tyrosine Hydroxylase Immunoreactive Neurons Which Is Maintained During Lactation

**DOI:** 10.3389/fendo.2018.00685

**Published:** 2018-11-22

**Authors:** Zsuzsanna Bardóczi, Tamás Wilheim, Katalin Skrapits, Erik Hrabovszky, Gergely Rácz, András Matolcsy, Zsolt Liposits, Joanna H. Sliwowska, Árpád Dobolyi, Imre Kalló

**Affiliations:** ^1^Laboratory of Endocrine Neurobiology, Institute of Experimental Medicine, Hungarian Academy of Sciences, Budapest, Hungary; ^2^School of Ph.D. Studies, Semmelweis University, Budapest, Hungary; ^3^Department of Neuroscience, Faculty of Information Technology, Pázmány Péter Catholic University, Budapest, Hungary; ^4^Laboratory of Reproductive Neurobiology, Institute of Experimental Medicine, Hungarian Academy of Sciences, Budapest, Hungary; ^5^1st Department of Pathology and Experimental Cancer Research, Semmelweis University, Budapest, Hungary; ^6^Laboratory of Neurobiology, Institute of Zoology, Poznan University of Life Sciences, Poznań, Poland; ^7^MTA-ELTE Laboratory of Molecular and Systems Neurobiology, Department of Physiology and Neurobiology, Hungarian Academy of Sciences and Eötvös Loránd University, Budapest, Hungary

**Keywords:** preoptic area, arcuate nucleus, tyrosine hydroxylase, kisspeptin, asymmetric synapses, estrogen, lactation, kisspeptin

## Abstract

Gonadotropin releasing hormone (GnRH) neurons provide neuronal input to the preoptic area (POA) and the arcuate nucleus (Arc), two regions involved critically in the regulation of neuroendocrine functions and associated behaviors. These areas contain tyrosine hydroxylase immunoreactive (TH-IR) neurons, which play location-specific roles in the neuroendocrine control of both the luteinizing hormone and prolactin secretion, as well as, sexually motivated behaviors. Concerning changes in the activity of GnRH neurons and the secretion pattern of GnRH seen under the influence of rising serum estrogen levels and during lactation, we tested the hypothesis that the functional state of GnRH neurons is mediated via direct synaptic connections to TH-IR neurons in the POA and Arc. In addition, we examined putative changes of these inputs in lactating mice and in mothers separated from their pups. Confocal microscopic and pre-embedding immunohistochemical studies on ovariectomized mice treated with 17β-estradiol (OVX+E2) provided evidence for direct appositions and asymmetric synapses between GnRH-IR fiber varicosities and TH-IR neurons in the POA and the Arc. As TH co-localizes with kisspeptin (KP) in the POA, confocal microscopic analysis was continued on sections additionally labeled for KP. The TH-IR neurons showed a lower level of co-labeling for KP in lactating mice compared to OVX+E2 mice (16.1 ± 5% vs. 57.8 ± 4.3%). Removing the pups for 24 h did not alter significantly the KP production in TH-IR neurons (17.3 ± 4.6%). The mean number of GnRH-IR varicosities on preoptic and arcuate TH cells did not differ in the three animal models investigated. This study shows evidence that GnRH neurons provide direct synaptic inputs to POA and Arc dopaminergic neurons. The scale of anatomical connectivity with these target cells was unaltered during lactation indicating a maintained GnRH input, inspite of the altered hormonal condition.

## Introduction

The gonadotropin-releasing hormone (GnRH) neurons are thought to form the final common pathway for central regulation of fertility by receiving input from phenotypically diverse neurons of several brain regions, and projecting to the median eminence, where their peptide hormone is released to reach the adenohypophysis. It is well-known, that cellular elements of the neuronal circuit regulating GnRH secretion receive indirect information about the secretory activity of GnRH neurons via hormonal feedback from the periphery; in contrast, it is less understood whether efferents of GnRH neurons can directly influence the hypothalamic neuronal circuitry that controls fertility. It has been established, that GnRH neurons innervate other GnRH neurons (1–3). More recently we have also shown that while GnRH processes project toward the median eminence, they establish synaptic connections with kisspeptin (KP) neurons of the POA, as well as, the arcuate nucleus (Arc) (4). These observations suggested the existence of signaling mechanisms that convey information about the activity of GnRH neurons to other neuronal systems involved in reproductive control. The potential physiological role of GnRH in the Arc was supported by its *in vitro* effect on the firing activity of non-identified neurons of Arc in slice preparation of rat brains (5).

The hypothalamic dopaminergic neurons maintain an inhibitory control on prolactin-secreting cells in the hypophysis (6); this tonic effect is important for the pulsatile secretion of GnRH, since a reduction of dopamine secretion during lactation results in an increased prolactin level, and consequently, suspension of the pulsatile secretion of GnRH (7, 8). However, this lactation-related infertility is diminished by the decreasing demand for breast-feeding of the pups, and the pulsatile secretion of GnRH neurons returns. Previous studies described GnRH varicosities in contact with dopamine-synthesizing, tyrosine-hydroxylase-immunoreactive (TH-IR) neurons in the Arc (9, 10). Mitchell et al. (10) also suggested an estrous-cycle dependent plasticity for these connecting profiles by reporting their presence only in pro-estrus and estrus, but not in diestrus (10).

These observations prompted us to study whether (1) GnRH neurons establish synaptic connections with DA-secreting, tyrosine hydroxylase (TH)-IR neurons in the preoptic area and/or arcuate nucleus, (2) GnRH axonal connections to dopamine- and/or KP producing neurons in the POA and Arc show alterations in lactating animals, compared to non-lactating mice, and to mothers separated from their pups.

## Materials and methods

### Animals

Adult cycling [2–3 months old, 25–30 g body weight (b.w.), *n* = 20] and postpartum, lactating (3–4 months old, 25–32 g b.w., *n* = 16) female CD1 mice (Charles River, Hungary) were housed under controlled lighting [12:12 h light-dark cycle; lights on at 07: 00 h, Zeitgeber time (ZT)0] and temperature (22 ± 2°C), with access to food and water *ad libitum*. Pregnant and postpartum mothers were individually housed. All studies were carried out with the permission from the Animal Welfare Committee of the Institute of Experimental Medicine (No.2285/003) and Eötvös Loránd University (PEI/001/37-4/2015) in accordance with legal requirements of the European Community (Decree 86/609/EEC). Surgery was performed on animals under deep anesthesia induced by an intraperitoneally injected cocktail of ketamine (25 mg/kg b.w.), xylavet (5 mg/kg b.w.), and pipolphen (2.5 mg/kg b.w.) in saline. The cycling animals were ovariectomised (OVX, day 0) and 7 days later (d7) implanted subcutaneously with a silastic capsule (ID 1.57 mm, OD 3.18 mm) containing 17β-estradiol (0.625 μg in 20 μl sunflower oil; OVX+E_2_) (11). Three days after implantation (d10), they were sacrificed. Uterine weight was significantly increased in these mice compared to the vehicle-treated group (OVX+E2, 123.7 ± 4.7 mg vs. OVX+Vehicle, 32.2 ± 5 mg, *P* < 0.001, the latter model was not used in this study). Eight of the lactating mice were deprived of pups on postpartum d10 (24 h before perfusion), whereas the other eight animals remained with their pups (litter size adjusted to 4 pups) until perfusion (12, 13). On postpartum d11, lactating and pup-deprived animals were perfused transcardially together with the OVX+E_2_ treated mice, their brains were removed and processed for KP, TH, and GnRH immunohistochemistry.

### Tissue preparation for confocal microscopy

The animals were perfused transcardially with phosphate-buffered saline (PBS; 0.1 M) containing 4% paraformaldehyde (PFA). The brains were post-fixed in 2% PFA/PBS solution for 24 h at 4°C, cryoprotected overnight in 25% sucrose and 25 μm thick coronal sections were cut on a freezing microtome. The sections were divided into three sequential pools and stored in antifreeze solution (30% ethylene glycol; 25% glycerol; 0.05 M phosphate buffer; pH 7.4) at −20°C until use.

After the endogenous peroxidase activity had been quenched with 0.5% hydrogen peroxide (10 min), sections were permeabilized with 0.5% Triton-X-100 (23,472–9, Sigma-Aldrich; 20 min). Finally, 2% normal horse serum (NHS) was applied (20 min) to reduce non-specific antibody binding. Subsequent treatments and interim rinses in PBS (3 × 5 min) were carried out at room temperature, except for incubation in the primary antibody or fluorochrome conjugate, which took place at 4°C.

### Tissue preparation for electron microscopy

In order to investigate putative GnRH-TH appositions in the POA and Arc at the electron microscopic level, pre-embedding, double-label immunohistochemistry was performed. For tissue preservation of estrogen-treated OVX animals (*n* = 5), a mixture of 2% PFA and 4% acrolein was used. 30 μm thick coronal sections were cut with a vibratome and treated with 1% sodium borohydride (30 min), 0.5% H_2_O_2_ (15 min) and permeabilized with three freeze-thaw cycles, as described previously (14).

### Electron microscopy to investigate the contacts between GnRH-IR fiber varicosities and TH-IR neurons

Sections from OVX+E_2_ mice containing the POA or Arc were double-labeled to detect GnRH and TH immunoreactivities. They were incubated for 48 h in a cocktail of antisera containing guinea pig anti-GnRH (#1018, Hrabovszky, 1:600,000) (15) and chicken anti-TH (#TYH, Aves Laboratories Inc., Tigard, OR, 1:1,000). Subsequently the GnRH-IR structures were visualized first by sequential incubation of the sections in biotinylated donkey anti-guinea pig IgG (#706-065-148, Jackson ImmunoResearch Laboratories, 1:1,000, 24 h) and Vectastain ABC Elite solution (1:1,000, 1.5 h). The peroxidase reaction was run in the presence of H_2_O_2_ and nickel-diaminobenzidine (NiDAB), and post-intensified with silver-gold (16). Following this reaction, the TH-IR structures were detected by incubating sections sequentially in biotinylated donkey anti-chicken IgG (#703-065-155, Jackson ImmunoResearch Laboratories, 1:1,000, 24 h) and Vectastain ABC Elite solution (1:1,000; 1.5 h). Then the peroxidase reaction was carried out in the presence of H_2_O_2_ and DAB alone. After completion of the immunohistochemical detection the sections were treated with 1% osmium tetroxide (1 h) and 1% uranyl acetate (in 70% ethanol; 40 min), dehydrated in an ascending series of ethanol and propylene oxide, and flat-embedded in TAAB 812 medium epoxy resin between glass microscope slides pre-coated with a liquid release agent (#70880; Electron Microscopy Sciences, Fort Washington, Pa., USA). The resin was allowed to polymerize for 2 days at 56°C. The flat-embedded sections were initially examined under a light microscope at 60 × magnification; areas exhibiting appositions of GnRH-IR processes on the somatodendritic region of TH-IR neurons within the POA and Arc were selected for further processing. Ultrathin (60 nm) sections were cut with a Leica ultracut UCT ultramicrotome (Leica Microsystems, Vienna, Austria). The ultrathin sections were collected in ribbons onto Formvar-coated single-slot grids, contrasted with 2% lead citrate and examined with a Jeol- 100C transmission electron microscope.

### Immunofluorescent triple labeling to detect GnRH, TH and KP in the POA and Arc

Every third sections from the POA and Arc regions of the OVX+E_2_, lactating and lactating pup-deprived animals were incubated for 72 h in a cocktail of the guinea pig anti-GnRH (#1018, Hrabovszky, 1:10,000)(15), rabbit anti-KP (#566, Caraty, 1:10,000)(17) and chicken anti-TH (#TYH, Aves Laboratories Inc., Tigard, OR, 1:1,000) primary antibodies. First, GnRH immunoreactivity was visualized with FITC-conjugated donkey anti-guinea pig IgG (#706-095-148, Jackson ImmunoResearch Laboratories, 1:1,000, 12 h). Then the KP- and the TH-IR structures were detected by incubating the sections in CY3-conjugated donkey anti-rabbit IgG (#711-165-152, Jackson ImmunoResearch Laboratories, 1:1,000, 2 h), and Cy5-conjugated donkey anti-chicken IgG (#703-175-155, Jackson ImmunoResearch Laboratories, 1:1,000, 2 h), respectively.

### Confocal laser microscopic analysis and 3-D reconstruction of GnRH-IR afferents to TH-IR neurons in the POA and Arc

Selected triple-labeled sections were scanned by using a Zeiss LSM780 confocal microscope. Matching sections from selected rostral, middle, and caudal levels of the POA (Bregma levels +0.25 to +0.13, +0.13 to −0.11 and −0.11 to −0.23 mm) and the Arc (Bregma levels −1.43 to −1.55, −1.67 to −1.79 and −1.91 to −2.03 mm) were identified from each animal and analyzed for the number of GnRH fiber varicosities contacting TH and/or KP-IR neurons. Multiple stacks of optical slices (1024 × 1024 pixels, z-steps 0.6 μm) were obtained by scanning all of the TH- and KP-IR neurons unilaterally in each of the selected coronal sections using a Plan Apochromat 63 x /1.4 NA oil immersion objective. The FITC, CY3 and Cy5 fluorochromes were detected with laser lines 488, 561, and 633 nm, using dichroic/emission filters 493–556 nm, 570–624 nm, and 638–759 nm, respectively. The sequentially recorded green, red and far-red channels were merged and displayed with the Zen software (Carl Zeiss). The images acquired with the confocal laser microscope were further investigated using three-dimensional (3-D) analyses. The stack of optical slices was loaded into the Amira 6.0 visualization software (Visual Imaging Group) and the two channels containing images of consecutive optical slices were rendered in three dimensions with surfaces generated from above-threshold immunoreactivity. The threshold was set individually for each image and color channel to minimize any noise, while maintaining the proper cellular boundaries. The surfaces generated from the two channels in the same optical volume were visualized to check for cell-to-cell contacts. This enabled verification of the findings from the two dimensional confocal image analyses.

### Experimental design and quantification process

OVX+E_2_ treated (*n* = 20) and lactating (*n* = 8) and pup-deprived (*n* = 8) animals were used to characterize the cellular interaction of GnRH axonal processes with TH-IR neurons in the POA and ARC. In order to better define anatomical regions, immunostained sections were compared with their matching counter-stained pairs. Efforts were made to minimize animal usage while ensuring adequate representation of the different rostro-caudal levels of the POA and ARC in the analyses. Thus, every fourth section (with 50 and 60 μm tissue gaps for confocal and electron microscopy, respectively) was processed for immunostaining; out of these, three sections per preoptic and arcuate regions were selected for quantitative analyses {[OVX+E_2_ treated (*n* = 5) and lactating (*n* = 4) and pup-deprived (*n* = 4) animals]}. Regions of interest (ROI; 50589 μm^2^) containing TH-IR neurons in the POA (3–6 ROI/Bregma levels to cover the entire area) and ARC (3–7 ROI/Bregma levels) were scanned (to a depth of 19–20 μm) in one side of the selected three sections of the POA or ARC regions. The regions analyzed in the rostral three sets of sections are called preoptic, within the distribution of GnRH fibers, KP neurons and the A14–15 TH cells can be found medially, with a large majority located in the periventricular area involving the anteroventral periventricular nucleus and caudally the periventricular hypothalamic nucleus. To avoid bias, Z-stacks were encoded and provided for analyses to an independent investigator blind to treatments. Each perikarya showing TH- and/or KP-immunoreactivity, and receiving GnRH afferent(s) has been recorded. Appositions (defined by the absence of any visible gap between the juxtaposed profiles in at least one optical slice) and immunoreactive perikarya were numbered. Both perikaryal and dendritic appositions were counted; dendrites were considered only if their connections to the perikaryon was traceable. To avoid double counting of perikarya or appositions, immunoreactive profiles appearing repeatedly in the overlapping parts of neighboring Z-stacks or neighboring optical slices of the Z-stacks were identified and encoded with the same number. KP-immunopositive or KP-immunonegative TH-IR cell populations, and TH-immunonegative KP-IR neurons were distinguished in the POA and named as KP^−^/TH^+^, KP^+^/TH^+^, KP^+^/TH^−^, respectively. In accordance with previous studies, the TH-IR neurons in the ARC were immunonegative for KP. The percentage of neurons immunoreactive either for TH or KP, or for both TH and KP, and the percentage of GnRH appositions on each of these neurons in the POA were determined and comparisons made for the different models by means of one-way ANOVA, with the *post hoc* Tukey HSD test. The mean number of GnRH appositions on the different phenotype of cells were also determined. This approach made comparisons between the preoptic and arcuate TH-IR cell populations possible by using two-way ANOVA, with the *post hoc* Tukey HSD. Statistical significance was defined at *p* < 0.05.

### Immunohistochemical controls

The specificities of the GnRH, TH, and KP primary antisera were reported previously (17–19). Negative controls included the use of increasing dilutions of the primary antisera, which resulted in a commensurate decrease and eventual disappearance of the immunostaining. Omission of the primary antibodies or their preabsorption with corresponding peptide antigens [(1 μM KP10 (NeoMPS, Strasbourg, France) for #566 antiserum(17)], resulted in complete loss of the immunostaining. Besides negative controls, positive controls were also carried out (by employing well-characterized reference antibodies) to validate the staining pattern generated by the GnRH and TH antibodies. Thus, two sets of sections were dual-immunolabeled by using the guinea pig anti-GnRH and the chicken anti-TH antisera with the following reference antibodies: rabbit anti-GnRH (LR1 from R. A. Benoit), mouse anti-TH (#22941 from Immunostar), respectively. By employing two different fluorochromes, the test antisera generated overlapping signals with the reference antibodies for each antigen. Secondary antibodies were designed for multiple labeling and pre-absorbed by the manufacturer with immunoglobulins from several species, including the one in which the other primary antibody had been raised.

## Results

### GnRH-IR fiber varicosities establish asymmetric synapses with TH-IR neurons in both the POA and Arc

**Figure 1:**
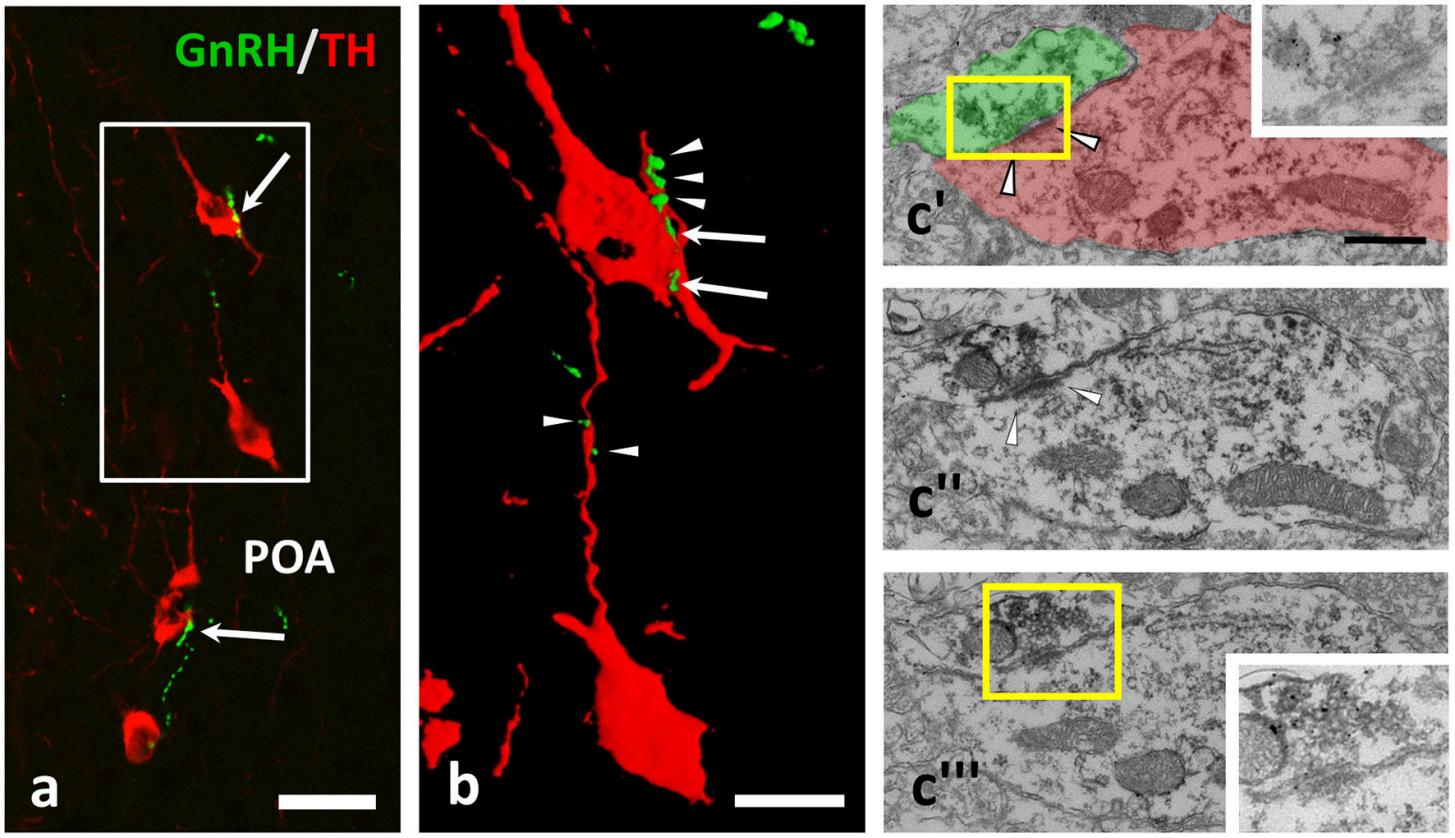
GnRH-immunoreactive (IR) beaded axons contacting processes of TH-IR neurons in the preoptic area of the mouse brain. **(a,b)** Double immunofluorescent labeling for GnRH (green) and TH (red) shows varicose GnRH-IR fibers in apposition to TH-IR cell bodies (arrows) and dendrites (arrowheads); the gap free connection was validated by reconstructing the image of the immunolabeled profiles in 3D **(b)**. Using medium electron dense DAB to label TH-IR neurons and highly electron dense SGI-NiDAB to mark GnRH-IR axons in pre-embedding immuno-electron microscopy, **(c)** asymmetric synapses (arrowheads) were identified. The presence of silver grains and the asymmetric character of the synapse were validated in consecutive ultrathin sections **(c**'–**c)**”'. Insets in **c**' and **c**”' demonstrate the boxed areas at higher power and a reduced contrast. Scale bar in **(a)** is 25 μm; in **(b)** it is 10 μm and in **(c**'–**c)**”' 500 nm.

**Figure 2:**
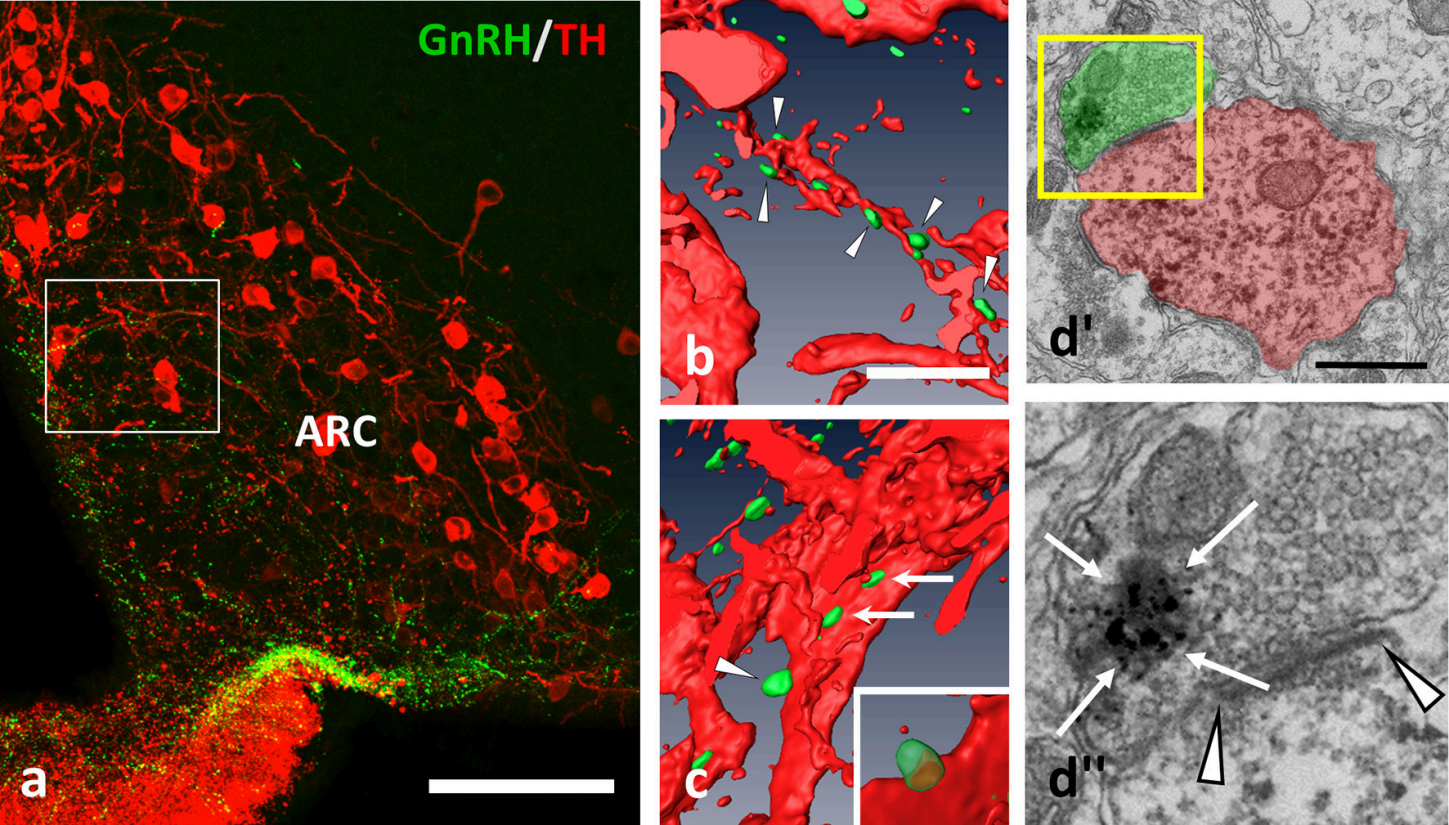
GnRH-IR varicosities (green) establish multiple appositions on TH-IR neurons (red) also in the Arc. **(a–c)** Axo-dendritic and axo-somatic appositions are indicated by arrowheads and arrows; **(b,c)** are snapshots from the 3D reconstructed and rotated image of the boxed area in **(a)**. The presence of asymmetric synapses **(d**; arrowheads) were confirmed also by the arcuate appositions. **(d)**” The GnRH-IR axon terminal contained both dense-core granules (arrows, heavily labeled with SGI-NiDAB) and round-shaped, small, clear vesicles, as shown in the high power image of the boxed area of **(d)**'. Scale bar in **(a)** is 100 μm; in **(b)** and **(c)** 10 μm; in **(d**'**)** it is 500 nm.

There are dopaminergic neurons in brain regions fundamental in the regulation of GnRH secretion forming the A14–15 group in POA, and the A12 group in the Arc. Immunohistochemical double labeling was carried out, which revealed GnRH-IR fiber varicosities establishing appositions onto TH-IR perikarya and dendrites in both the POA (Figures 1a,b) and Arc (Figures 2a–c) of OVX+E2 mice. Confocal microscopy and 3D reconstruction of the cellular profiles were used to validate the gap free connection of GnRH-IR varicosities with TH-IR neurons (Figures 1b, 2b,c). To study the ultrastructure of the contact sites, pre-embedding immunoelectron microscopy was employed, using silver-gold intensified (SGI)-NiDAB to mark GnRH processes and DAB to label the TH-IR neurons. At the electron microscopic level, the presence of synaptic specializations at the contact sites was confirmed for twelve cases. All of these showed asymmetric characteristics (Figures 1c, 2d) suggesting excitatory neurotransmission to dopaminergic neurons in both the POA and Arc.

To test the potential plastic changes of the GnRH input to dopaminergic neurons in lactating mothers (postpartum day 11) and in mothers deprived from pups for 24 h, immunohistochemical triple labeling (for GnRH, TH, and KP) and confocal microscopic analyses were conducted. The numbers of GnRH-IR fiber appositions to KP positive, as well as, KP negative TH-IR neurons (KP^+^/TH^+^-IR and KP^−^/TH^+^-IR) were analyzed in lactating mothers and compared to values shown by non-lactating (OVX+E2) mice, and mothers deprived from their pups for 24 h (Table 1).

**Table 1:** Number of neurons immunoreactive (IR) for tyrosine hydroxylase (TH) and/or kisspeptin (KP) in the preoptic area (POA) and gonadotropin releasing hormone (GnRH)-IR afferents on TH- and KP-IR neurons in the POA and on TH-IR neurons of the arcuate nucleus (Arc).

	**OVX**+**E2 (*****n*** = **5)**					**Lactating (*****n*** = **4)**					**Pups deprived (*****n*** = **4)**				
**Counted in ROIs**	**All IR**	**KP^−^/TH^+^**	**KP^+^ /TH^+^**	**All TH+**	**KP^+^ /TH^−^**	**All IR**	**KP^−^/TH^+^**	**KP^+^ /TH^+^**	**All TH+**	**KP^+^ /TH^−^**	**All IR**	**KP^−^/TH^+^**	**KP^+^ /TH^+^**	**All TH+**	**KP^+^ /TH^−^**
Neurons in the POA	1317	106.2 ±15.8	150.6 ±10.8	256.8 ±15.9	6.6 ±2.7	1084	189.5 ±33	41.2 ±12.8	230.7 ±31.5	40.2 ±11.9	1068	184.7 ±26.6	48.7 ±17.3	235.5 ±41	33.5 ± 8.8
Neurons in the Arc	935	187 ±13	N/A	187 ±13	N/A	717	179.2 ±17.2	N/A	179.2 ±17.2	N/A	737	184.2 ±7.1	N/A	184.2 ±7.1	N/A
GnRH appositions on neurons in the POA	370	37.2 ±8.8	34.8 ±3	72 ±11.7	2 ±1	213	37.7 ±10.7	5.7 ±1.4	43.5 ±9.8	9.7 ±3	188	36 ±10.9	6 ±2.8	40.7 ±12.2	5 ±1.4
GnRH appositions on neurons in the ARC	413	82.6 ±5.8	N/A	82.6 ±5.8	N/A	272	68 ±11	N/A	68 ±11	N/A	247	61.7 ±6.2	N/A	61.7 ±6.2	N/A

*TH- and KP-IR neurons and GnRH-IR appositions were counted in one side of three selected POA and ARC sections and given in the form of absolute numbers (‘all IR'; the sum of all counted items) and Mean ± SEM for each experimental model*.

### Lower ratio of KP^+^/TH^+^neurons in the POA of lactating mothers

**Figure 3:**
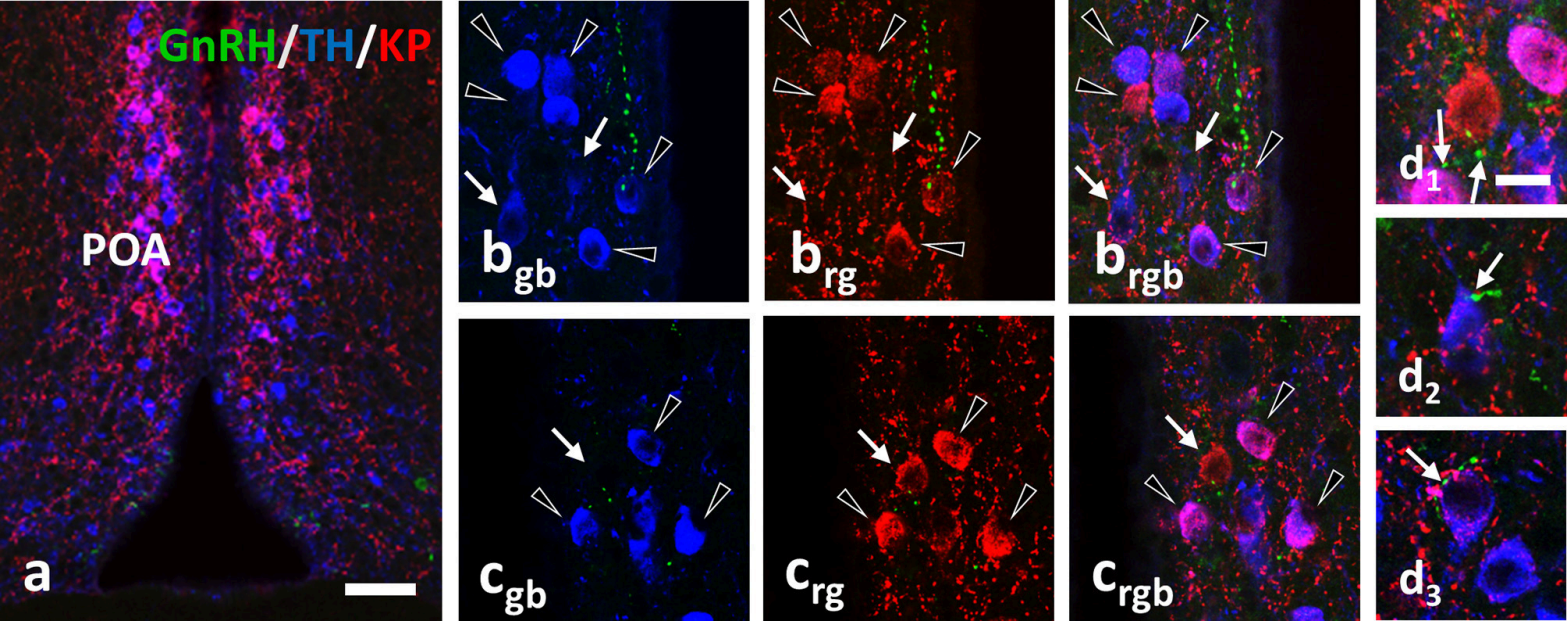
Immunohistochemical triple labeling for GnRH (green), TH (blue) and KP (red) in the POA, where the majority of TH neurons are also immunoreactive for KP **(a**; co-localization in purple). Besides double-labeled neurons (arrowheads), single-labeled for TH (arrows in **b)** or KP (arrow in **c)** are also present. The GnRH-IR varicosities form appositions (arrows) on all three phenotypes of neurons **(d1–d3)**. Scale bar in **(a)** is 50 μm; in (bgb–**d3)** 10 μm.

A relatively high percentage of TH neurons was found to be immunoreactive for KP in OVX-E2 mice (Figure 3, and Figure **5a**; 57.8 ± 4.3%). This ratio was significantly lower in lactating mice (16.1 ± 5%, of all IR cells counted, *F* = 28.069, *p* < 0.001; one-way ANOVA, *post hoc* Tukey, Figure **5a**). The percentage of neurons single-labeled either for TH (KP^−^/TH^+^-IR) or KP (KP^+^/TH^−^-IR) was in turn significantly elevated in lactating mice (for TH; 39.8 ± 3.7% vs. 72.4 ± 2.9% of all IR cells counted, *F* = 30.986, *p* < 0.001; one-way ANOVA, *post hoc* Tukey, for KP; 2.4 ± 0.8% vs. 15.4 ± 4.1% of all IR cells counted, *F* = 5.056, *p* = 0.03; one-way ANOVA, *post hoc* Tukey, Figure **5**). Removing the pups from the litter for 24 h did not change the co-localization percentages in the mothers (17.3 ± 4.6% of all IR cells counted; Figure **5a**).

### Effect of lactation or pup-deprivation on the number of GnRH-IR fiber appositions onto TH-IR neurons

**Figure 4:**
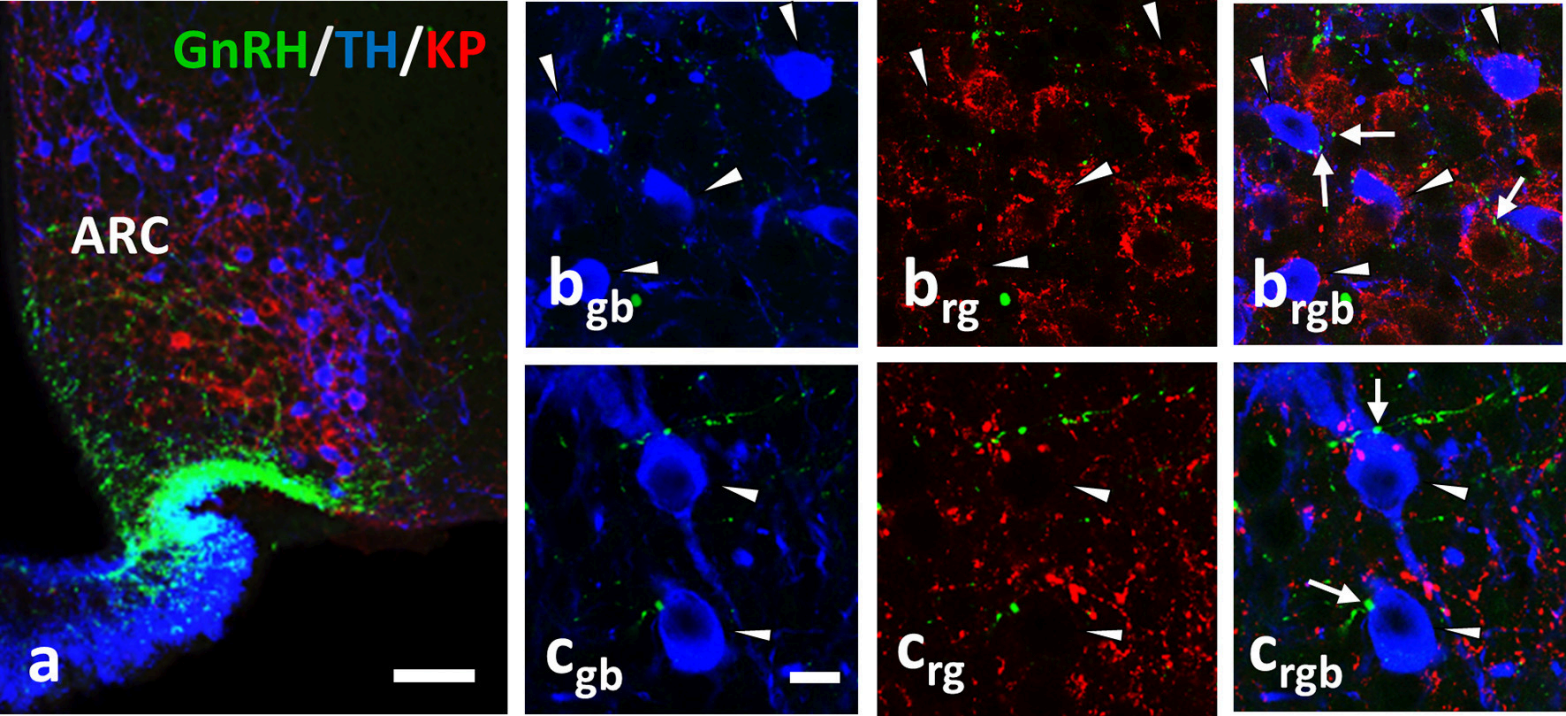
Immunohistochemical triple labeling for GnRH (green), TH (blue) and KP (red) in the Arc, where TH neurons are not immunoreactive for KP **(a)**; TH- and KP-IR neurons form two separate populations. The TH-IR neurons (arrowheads) receive axo-somatic and axo-dendritic appositions from GnRH-IR fibers (arrows; **b,c**). rgb; red-green-blue channels. Scale bar in **(a)** is 50 μm; in **(b**_gb_**-c**_rgb_**)** 10 μm.

**Figure 5:**
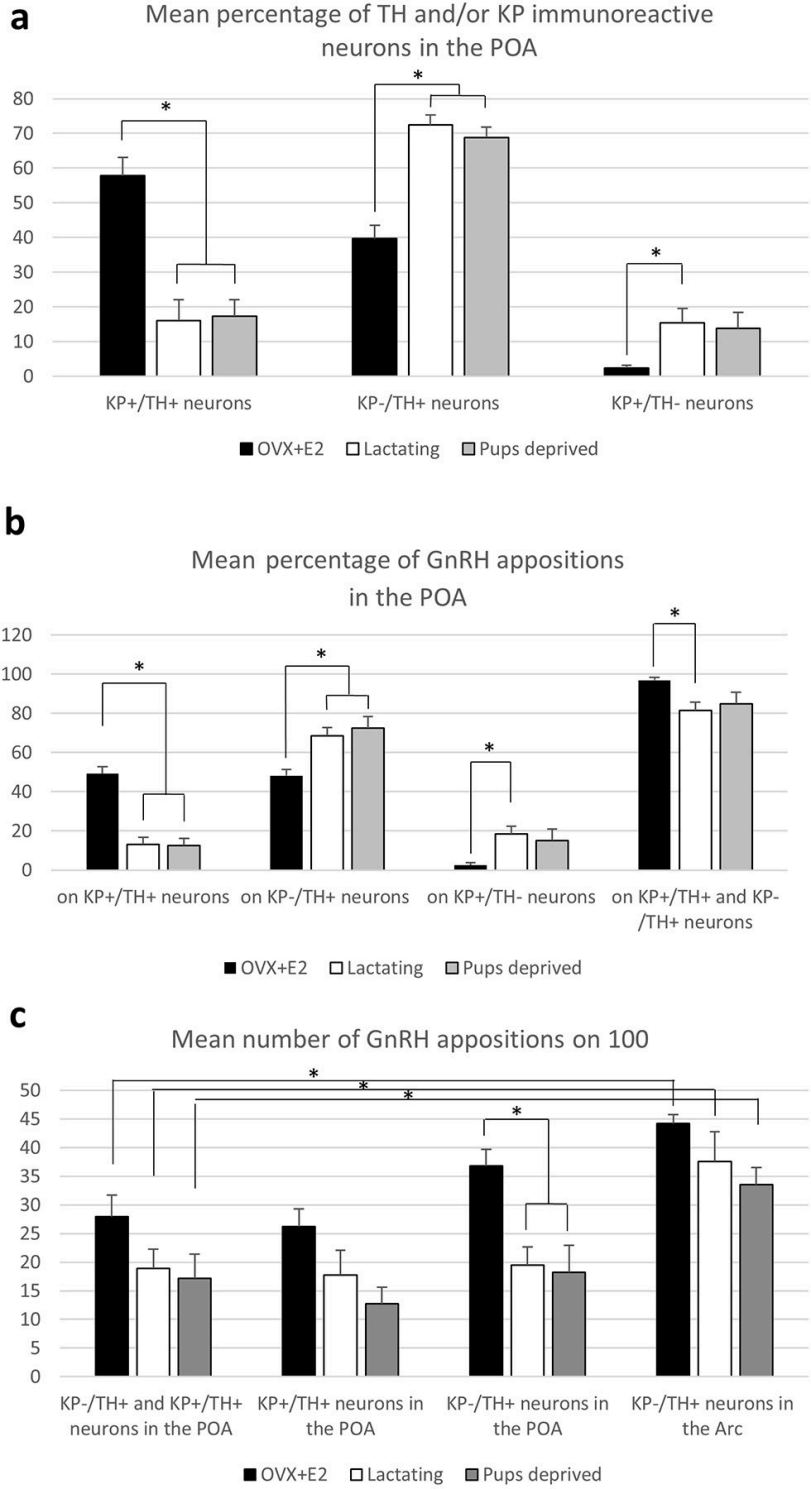
Characterization of preoptic and arcuate TH-IR neurons for kisspeptin expression and GnRH-IR afferentation. **(a)** Percentage of neurons immunoreactive (IR) for tyrosine hydroxylase (TH) and/or kisspeptin (KP) in the preoptic area (POA). **(b)** Percentage of the GnRH appositions on each of this neuronal phenotype in the POA. **(c)** The mean number of GnRH appositions on 100 TH-IR neurons in the POA and arcuate nucleus (Arc). Asterisks mark significant changes.

Using confocal microscopic analyses, varicose GnRH-IR fibers were observed in apposition to all three phenotypes of labeled neurons (i.e., KP^+^/TH^+^, KP^−^/TH^+^, or KP^+^/TH^−^) in the POA (Figure 3) and to the TH-IR neurons (i.e., KP^−^/TH^+^) in the Arc (Figure 4). Mothers showed a significantly elevated percentage of GnRH-IR appositions to KP^−^/TH^+^ neurons in the POA (68.5 ± 4.3% for lactating mice, 72.3 ± 6% for pup-deprived mothers vs. 48.2 ± 3.2% for the OVX+E2 mice, *F* = 8.834, *p* = 0.006; one-way ANOVA, *post hoc* Tukey; Figure 5b). In contrast, KP^+^/TH^+^ neurons received a significantly reduced GnRH innervation in the same groups of animals (13.1 ± 3.8% and 12.4 ± 3.7% vs. 49.3 ± 3.4%, respectively, *F* = 36.225, *p* < 0.001; one-way ANOVA, *post hoc* Tukey; Figure 5b). The net result was a small reduction in the percentage of GnRH appositions on the entire population of preoptic TH-IR neurons of lactating female mice compared to non-lactating animals (*F* = 4.946, *p* < 0.032; one-way ANOVA, *post hoc* Tukey; Figure 5b). Removing the pups from the litter caused no significant changes in the number of contacts. The percentages of GnRH appositions targeting the TH-IR neuron population (KP^+^ and KP^−^) were high (96.9 ± 1.5%, 81.6 ± 4.1%, 84.8 ± 5.9% in the POA of OVX+E2 mice, lactating or pup-deprived mothers, respectively; Figure 5b). The mean number of GnRH appositions per TH-IR neuron did not differ among the experimental groups, but its value was significantly lower in the POA than in the ARC for each experimental group (*F* = 34.043, *p* < 0.001; two-way ANOVA, *post hoc* Tukey; Figure 5c).

## Discussion

Dopaminergic neurons, participating in the regulation of prolactin secretion, integrate multiple signals to provide lactotrophs with a tonic inhibitory input (20). The inhibitory signal transmission is predominantly activated via the short-loop feedback effect of prolactin itself, but neuronal afferents may also contribute to the regulation of dopaminergic neurons (6). The neuronal inputs can be supplementary in function to keep prolactin levels low, but may also exert opposite effects to facilitate prolactin secretion at late pregnancy and/or during lactation.

**Figure 6:**
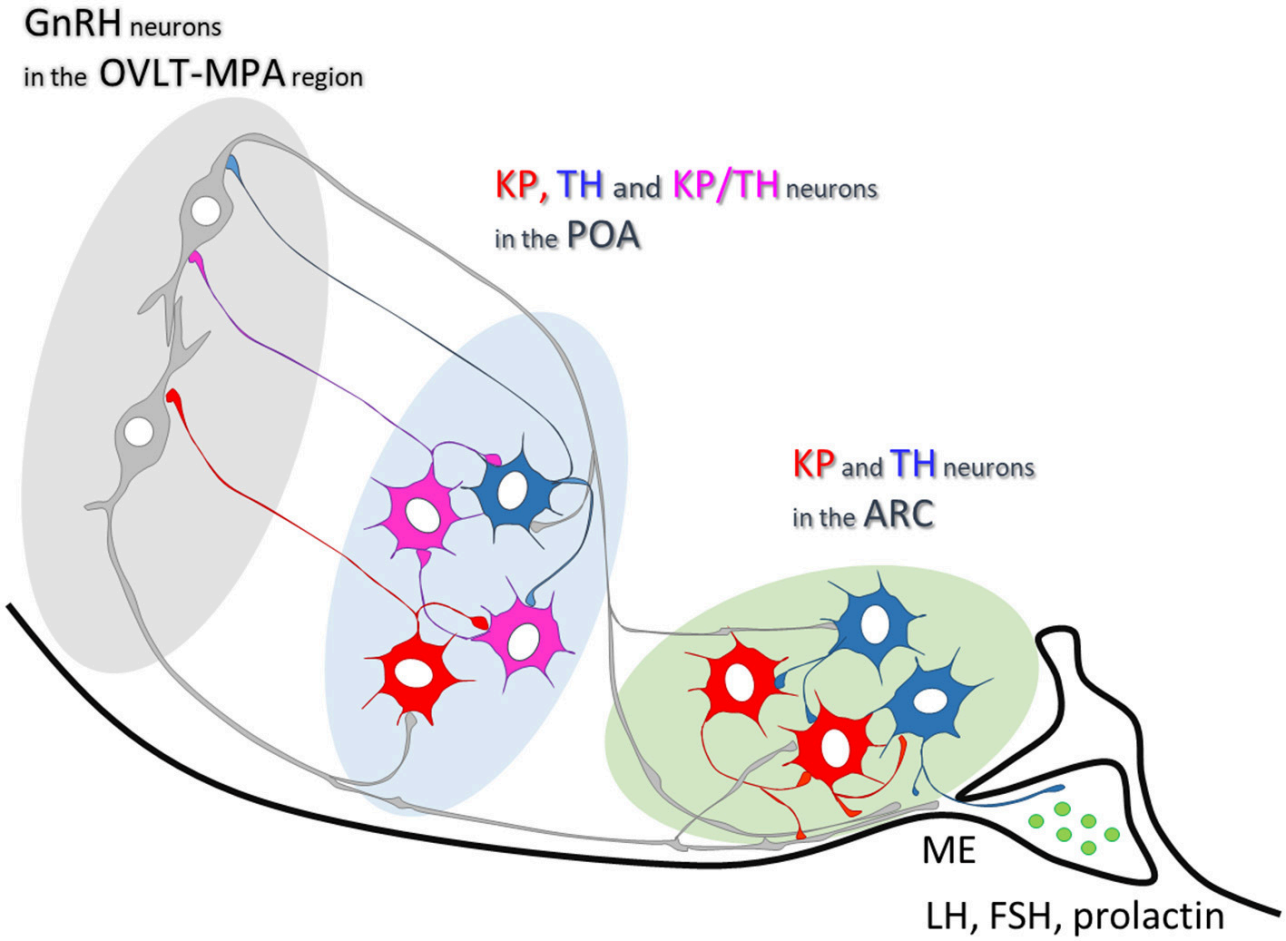
Summary scheme illustrating neuronal interactions between GnRH and dopaminergic neurons in mice. In the mouse preoptic area, about two-thirds of TH-IR neurons are also immunoreactive for KP. One-third of them, similarly to the Arc dopaminergic neurons, are distinct from KP-IR neurons. Axonal branches of GnRH processes establish synaptic connections with the TH- and KP-IR cell populations in both the preoptic area and arcuate nucleus.

In the current study, we demonstrate GnRH afferents to TH-IR neurons located in the mouse periventricular region of the POA, as well as, in the mouse arcuate nucleus (see for summary diagram in Figure 6). This supplements our previous data showing similar connection of GnRH axon varicosities with preoptic KP-IR cells (4). Considering that almost all preoptic KP neurons express TH (21) (more than 90% in the current study) the question has emerged, whether only KP-expressing TH neurons are targets of GnRH afferents. Although the percentage of preoptic KP^−^/TH^+^ neurons varied in the different animal models, they represented nearly half of all immunoreactive neurons in the OVX+E2 animals, and more than two-third of all immunoreactive neurons in lactating and pup-deprived mothers (Figure 5a). Relatively high percentages of these neurons received GnRH-IR fiber appositions in all experimental models Figure 5b, indicating that the KP immunonegative TH-IR neurons represent a second major neuronal population in the POA targeted by GnRH afferents. The TH-IR neurons in the Arc, where they form a completely separate cell population from KP neurons (21, 22), were also found to receive input from GnRH axon terminals (Figures 2, 4, 6).

As GnRH neurons are phenotypically heterogeneous, the question arises which subpopulation of GnRH neurons innervates the dopaminergic neurons. Dumalska et al. (23) reported that in VGLUT2-GFP mice 84% of GnRH-immunoreactive neurons expressed GFP; moreover, the cytoplasmic extract of each GnRH-GFP cell recorded, also contained mRNA for VGLUT2. These data indicated the glutamatergic character of most GnRH neurons in mice, 61% of which also responded to kisspeptin, but not to group I metabotropic glutamate receptor agonists in their *in vitro* study (23). In contrast, Zhu et al. (24) observed GFP expression in 62% of GnRH-immunoreactive neurons in GAD67-GFP mice indicating that more than half of the GnRH neurons produce GABA, as a neurotransmitter. In addition, they demonstrated a sex and hormonal dependent variation in the ratio of GABAergic GnRH neurons (24). The asymmetric type of synapses found exclusively between GnRH axons and TH-IR neurons in the current study suggests that the GnRH neurons innervating the TH-immunoreactive neurons use glutamate, and very likely exert excitatory effects on these dopaminergic neurons. Nevertheless, the possibility for GABA to appear in the GnRH axon terminals synapsing on TH-IR neurons cannot be excluded. The non-complementary ratio of glutamatergic and GABAergic GnRH (82–100% *vs*. 64%) neurons found by Dumalska and Zhu, respectively, at least raises the possibility for some GnRH neurons to exhibit a double phenotype for classical neurotransmitters, similarly to other preoptic neurons reported before (25). Further studies are needed to clarify whether only glutamate or glutamate together with GABA participate as classical transmitters in the synaptic communication of GnRH and dopaminergic neurons.

The current study focused on the GnRH neuronal projections to the preoptic (A14–15) and the arcuate (A12) subgroups of the dopaminergic neurons. These subpopulations of dopaminergic neurons establish local connections, as well as, project to the median eminence (TIDA cells), or the posterior and intermediate lobes of the pituitary gland (THDA and PHDA), where they access the short portal vessels to transport dopamine to the anterior pituitary gland (26). As far as the GnRH afferents to these dopaminergic neurons are concerned, we found no preferential targeting of GnRH axons to any one of these three subpopulations. This is reminiscent to the prolactin receptor expression in all three subgroups of dopaminergic cells (27), which indicates that these neurons may contribute similarly to the regulation of prolactin secretion.

Based on the asymmetric type of the synapses established, it is reasonable to think that the GnRH neuronal afferents stimulate the dopaminergic cells under certain conditions and facilitate dopamine secretion; consequently, they inhibit prolactin secretion from the pituitary gland. This effect would be in congruence with the biological need of the normal estrous cycle to keep the inhibitory effect of prolactin low on GnRH secretion. However, secretion of prolactin is far more complicated, showing pro-oestrus and lactation related surges, the regulation of which cannot be directly associated with the transmitter-release of GnRH neurons.

Mitchell et al. (10) reported an estrous cycle dependent variation in the number of contacts between GnRH processes and TH-IR neurons in the Arc, suggesting a hormone-dependent plasticity for the communication (10). A profound suppression of tyrosine hydroxylase mRNA expression (28, 29), and phosphorylation (30, 31) of this enzyme with activity reduction have been shown during lactation. This might have contributed to the lower levels of preoptic neurons immunoreactive for both TH and KP, as well as, the lower percentage of GnRH appositions on the entire population of TH-IR neurons in lactating vs. OVX+E2 mice. Furthermore, NPY, ENK and NT-immunoreactivities are enhanced in TIDA neurons during lactation, and removing the pups from the litter resulted in a marked depletion of the immunoreactivity for these peptides from the ME TH-IR endings (32) with a concurrent elevation of TH mRNA expression in the Arc (28). These observations prompted us to study, whether lactation, when the pulsatile secretion of GnRH is suspended, or removal of the pups and consequently the stimulus of breast-feeding reflexes for 24 h, induce plastic changes in the GnRH-TH connection. We found a significant increase in the percentage of GnRH apposition on single-labeled TH-IR (KP^−/^TH^+^) neurons in the POA of mothers, which was accompanied by the significantly reduced percentage of these afferents to KP^+^/TH^+^ neurons in the same groups of animals. However, when the mean number GnRH-IR appositions was investigated on the full population of preoptic (KP^−^ and KP^+^) and arcuate TH-IR neurons, no significant group difference could be observed (Figure 5c). This indicates, that the GnRH input of TH-IR neurons is maintained in the POA during lactation, while a subpopulation of the neurons shows a reduced KP expression, as it was reported before (33, 34). Similarly, no significant difference could be observed in the GnRH input of ARC neurons among the different experimental groups. However, the possibility cannot be excluded that plastic changes may occur at ultrastructural and/or molecular levels, at different time points of lactation or following a longer pup-deprivation.

Mitchell found GnRH-IR fiber varicosities in apposition to dopaminergic neurons in pro-estrous and estrous, but not in diestrus mice. Therefore, in the current study we used ovariectomised mice with estradiol replacement that mimics pro-estrous levels of estradiol and induces repetitive daily LH surges (11). This non-lactating model was opposed by the model of lactating females at *post partum* day 11, with reportedly low gonadotropin and estrogen levels in rodents (35, 36).

The relatively persistent high E2 levels in our OVX+E_2_ model resulted in a very high co-localization level between TH and KP immunoreactivities in the POA region; about 60% of all dopaminergic neurons were immunoreactive for KP. In accordance with previous studies (33, 37), these values were significantly lower in lactating females, indicating a reduced KP expression, not only in the Arc, but also in the POA. Contrasting the diestrous-like levels of estradiol, and the reduction of KP expression in TH neurons of lactating mice, the number of GnRH-IR varicosities contacting the dopaminergic neurons showed no significant changes in these animals.

In summary, by demonstrating contacts and asymmetric synapses between GnRH-IR processes and TH-IR neurons with confocal and electron microscopic approaches the data presented here provide the first direct morphological evidence for the GnRH-IR innervation of TH neurons in both the POA and the Arc. The present findings add the dopaminergic neurons to the list of GnRH neuron target cells. Expressing KP in a subset of cells in the POA, and lacking this feature in the Arc, TH neurons are postsynaptic targets of GnRH neurons, which enables them to perceive changes in the firing and/or secretory activity of GnRH neurons at various physiological conditions. How this capability of dopaminergic neurons is translated to secretion of gonadotrophs and/or lactotrophs or the reproduction and/or lactation-related behaviors is not known, yet. The maintenance of GnRH afferents to TH neurons during lactation indicates a rather consolidated character of this connection, which remains wired for the time when the pulsatile secretion of GnRH is temporarily suspended.

## Author contributions

IK supervised the work. All authors were involved in conceiving of the experiments in this study. ZB and IK wrote the MS with support from JHS, ZL, and EH. Tissue samples were provided by EH, GR, AM, and ÁD, whereas the confocal and electron microscopic experiments were carried out by ZB, KS and IK. TW generated the 3D reconstruction of immunostained cells and provided quantitative analyses.

### Conflict of interest statement

The authors declare that the research was conducted in the absence of any commercial or financial relationships that could be construed as a potential conflict of interest.

## Abbreviations

Arc: arcuate nucleus
DA: dopamine
E_2_: 17-β estradiol
ENK: encephalin
GnRH: gonadotropin-releasing hormone (GnRH)
IR: immunoreactive
KP: kisspeptin
KP^−^/TH^+^: neurons immunolabeled only for TH
KP^+^/TH^+^: neurons immunolabeled for both KP and TH
KP^+^/TH^−^ neurons immunolabeled only for KP
LH: luteinizing hormone
ME: median eminence
MPA: medial preoptic area
NPY: neuropeptide Y
NT: neurotensin
OVLT: vascular organ of the lamina terminalis
OVX: ovariectomized
PHDA: periventricular hypophysial dopaminergic neurons
POA: preoptic area
THDA: tuberohypophysial dopaminergic neurons
TIDA: tuberoinfundibular dopaminergic neurons
TH: tyrosine-hydroxylase.
